# Microwave modes of ambidextrous helices

**DOI:** 10.1038/s41598-025-15042-4

**Published:** 2025-08-19

**Authors:** J. Gudge-Brooke, N. Clow, A. P. Hibbins, A. W. Powell, J. R. Sambles

**Affiliations:** 1 Department of Physics and Astronomy, Centre for Metamaterial Research and Innovation, University of Exeter, Stocker Road, Exeter, EX4 4QL UK; 2 DSTL, Porton Down Salisbury, Wiltshire, SP4 0JQ UK

**Keywords:** Physics, Applied physics

## Abstract

This work presents an investigation into the electromagnetic properties of ambidextrous helices, structures composed of equal numbers of left- and right-handed turns, operating in the gigahertz frequency range. Unlike conventional single-handed helices, which support only hybridized, elliptically polarized modes, ambidextrous helices exhibit two distinct linearly polarised resonant modes: one with electric dipole-like behaviour and the other with magnetic dipole-like behaviour. Using a combination of numerical simulations and experimental validation, we analyse the two lowest-order modes of a four-turn ambihelix, highlighting their unique characteristics. A key result of this study is the demonstration that these resonances can be independently tuned through geometric modifications. By adjusting the pitch, the frequencies of the electric and magnetic dipole modes can be exchanged in frequency order and indeed made to be coincident. These findings underscore the ambihelix’s potential for reconfigurable and multifunctional microwave devices. Its dual-mode capability, combined with geometric control over resonance order and frequency, offers new design flexibility for compact antennas, metamaterials, and polarisation-sensitive applications. The four-turn ambihelix studied here represents just one example from a broader design space, highlighting the rich potential of ambidextrous helices for future electromagnetic engineering.

## Introduction

Electromagnetic helical resonators have been widely used in various applications, including wireless power transfer and antennas, and have been the focus of extensive research and development, particularly since Kraus’ influential work in 1947^1^. Helical antennas are generally considered to operate in one of two modes: the normal mode and the axial mode^2^. In actuality all modes are inevitably a mix of both because of the symmetry of the helix. Axial-mode helical antennas are commonly used in satellite communication and other applications where directional radiation is desirable with significant research attention directed towards this mode of operation^3–5^. In the normal mode, the helix behaves like a short dipole-antenna, with the current oscillating along the length of the helix. The radiation pattern is broad and typically oriented perpendicular to the axis of the helix. This normal-mode, has received some attention^6,7^ but still holds untapped potential with unexplored variations to their design and operation.

Helical antennas operating in the normal mode generally have dimensions (height, circumference) significantly smaller than the operating wavelength (unlike the axial mode). Despite their small physical size compared to operational wavelength, the fundamental mode of these antennas demonstrates a radiation pattern similar to that of a straight, half wavelength electric dipole antenna. Further, because of the circumferential currents the fundamental mode has a magnetic dipole as well as an electric dipole. A single handed helix will always produce elliptically polarised radiation with appropriate dimensions the electric field strength associated with the magnetic dipole could be made equal to that due to the electric dipole and thus, because of the 90 degree phase difference, circularly polarised radiation can be generated^8^. Circularly polarised radiation finds applications in mobile and satellite communication, RFID technology, and medical devices^9–12^.

Very little work has been undertaken on a simple variant of the standard uniform helix, that of an ambidextrous helix, where a number of right-handed turns are joined to an equal number of left-handed turns of the same pitch (this variant is labelled for convenience an “ambihelix”). This variant has been mentioned in early helical work^13^ although it was not pursued at the time. Bush et al^14^ demonstrated the efficacy of introducing a helical element with two arms of different handedness to increase far-field radiation of a compact antenna by minimising the magnetic field contribution. This structure is centre-fed at the junction between the left- and right-handed sections, constraining it to support only a limited set of symmetric current modes. There has also been work done on two handed helices in a spherical shape though the proprieties that arise from the two handedness were not investigated^15^. Many of the basic properties of ambihelices remain unexplored.

In this study, an in-depth analysis is conducted of the two lowest-order resonant modes of a four-turn ambihelix with comparison to a four-turn single-handed helix. By comparing their electromagnetic responses, key differences in the resonant behaviour are identified, focusing on the unique modal characteristics of the ambihelix. In particular, this work highlights the emergence of distinct electric and magnetic dipole-like modes in the ambihelix, and how these can be independently tuned through geometric modifications. This contrasts with the single-handed helix, whose resonant modes are of mixed character who with a fundamental and higher order harmonics.

This comparative approach highlights the potential for electromagnetic applications such as inductive charging^16^ using a near pure magnetic dipole that is enabled by the ambihelix dual-handed structure.

## Lowest order modes

### Geometry

**Fig. 1:**
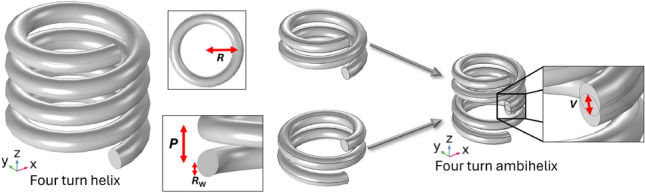
Single handed helix geometry (left) and construction of an ambihelix of two left handed turns and two right handed turns, highlighting the overlap region (right), *R* is the turn radius from axial centre to wire centre, $R_\textrm{W}$ is the wire radius, *P* is the pitch and *V* is the overlap.

To set the work in context we first explore a single-handed four-turn helix as a reference. Throughout we set the helix geometry for modelling and experiments to be formed from uncoated copper wire of diameter $2R_\textrm{W}$ = 1.7 mm with a helical radius of *R* = 4.0 mm, shown in the left-hand section of Fig. 1. *P* ,the pitch, is defined as the distance along its central axis corresponding to one complete 360-degree turn of the helix. The ambihelix is formed when two helices of different handedness are joined at the centre. In the experiment this join is a sharp bend while in the models there is a small overlap of *V* = 0.5 mm between the centre of wires of the different handed parts as shown in Fig. 1.

### Uniform single-handed helix lowest order modes

**Fig. 2:**
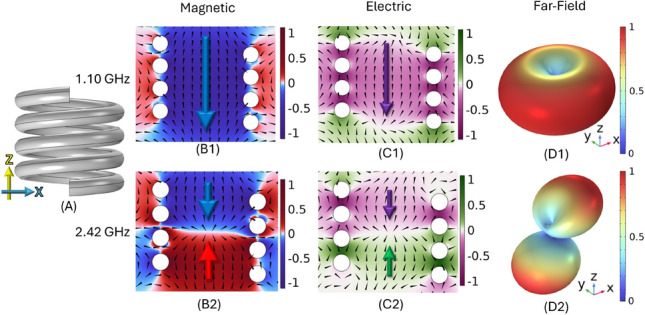
Two lowest order eigenfrequencies for a single-handed 4 turn, *P* = 2 mm , *R* = 4.0 mm, $R_\textrm{W}$ = 0.85 mm helix. Geometry (**A**), normalised magnetic fields ($B_z$) with field arrows (**B1, B2**), normalised electric field ($E_z$) temporally $\pi /2$ phase shifted from the magnetic field with fields arrows (**C1, C2**) of the lowest order modes in the zx-plane with far field radiation pattern (**D1, D2**).

In Fig. 2 the reference normal helix is shown alongside the radiation fields of both its fundamental and second-order modes obtained using a finite-element method (FEM) numerical model^17^ using an eigenfrequency solver. The top half of the figure illustrates that, in the fundamental mode, the electric and magnetic dipoles induced are nearly parallel to the helix’s axis and exhibit almost perfect dipolar behaviour, despite the chirality of the helix. Note that this first mode is exclusively dipolar, with no higher-order multipolar contributions.

In contrast, the second-order mode at around twice the frequency of the fundamental is quadrupolar. The radiation pattern is dominated by electric and magnetic quadrupoles rather than dipoles. Predictions of these quadrupole fields are shown in lower half of Fig. 2, the quadrupole mode radiation is far weaker than the dipolar mode.

The asymmetry observed in the linear quadrupole mode of the helix arises from the spatial differences between the electric and magnetic field distributions. Specifically, the zero-field regions at the centre of the helix differ for each field type: the magnetic field’s null line in the centre passes between adjacent turns, while the electric field’s null line intersects the centre of the turns on either side. This offset in the field geometries leads to a deviation from the idealised figure-of-eight far-field radiation pattern, resulting in a more complex and asymmetric emission profile as well as complex polarisations.

One may estimate the resonant frequencies of helical resonators using the total length of the wire:

1 $$\begin{aligned} L= & N_{turns} \sqrt{(2\pi R)^2 + P^2} \end{aligned}$$

2 $$\begin{aligned} f_1= & \frac{c}{2L} \end{aligned}$$

3 $$\begin{aligned} f_2= & \frac{c}{L} \end{aligned}$$

These predict the first two resonant frequencies to be approximately 1.49 GHz and 2.97 GHz. These values are higher than those obtained through simulation, primarily due to end effects and the slowing of electromagnetic waves along the helix. While this approach provides a useful first-order estimate that supports the validity of the simulated and experimental results, it is not sufficiently accurate for precise predictions in this specific geometry or for ambihelices.

### Lowest order modes of the Ambihelix

We now turn our attention to a four turn (two right handed, two left handed) ambihelix.

By combining two helical sections with opposing handedness, the helical structure gains mirror symmetry about its central horizontal plane, giving rise to distinct properties. When a voltage is applied between the ends this produces a uniform current where the magnetic fields generated by the two halves of the structure would oppose each other, resulting in cancellation of the magnetic contribution in the far-field.

**Fig. 3:**
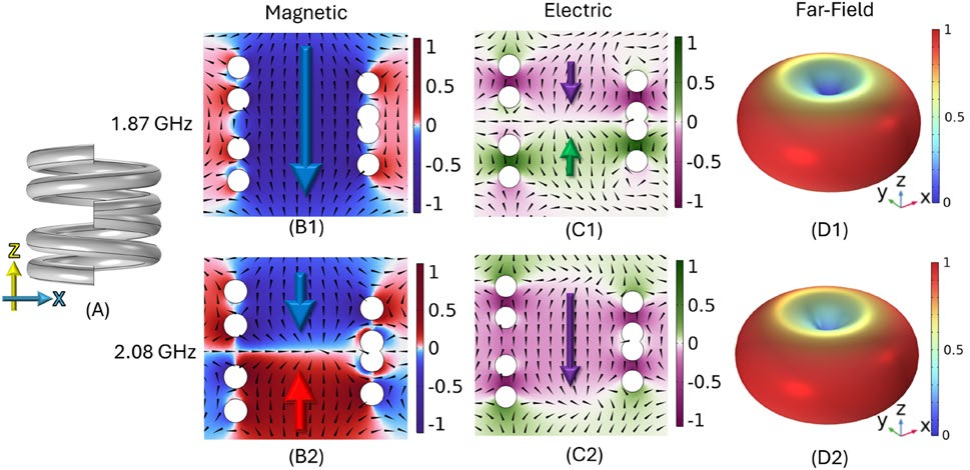
Two lowest order eigenfrequencies for a two left two right turn, *P* = 2 mm , *R* = 4.0 mm, $R_\textrm{W}$ = 0.85 mm ambihelix, *V* = 0.5 mm. Geometry (A), normalised magnetic fields ($B_z$) with field arrows (B1, B2), normalised electric field ($E_z$) temporally $\pi /2$ phase shifted from the magnetic field with fields arrows (C1, C2) of the lowest order modes in the zx-plane with far-field radiation pattern (D1, D2).

Consequently shown in the lower half of Fig. 3 the other mode has an electric dipole and a linear magnetic quadrupole making it a promising candidate for a compact, low-frequency electric dipolar antenna due to minimal perturbation in the far-field from the magnetic component as well as linear polarisation.

In contrast the mode which has a current distribution similar to that of a second order mode in a single handed helix, produces a magnetic dipole-like mode as shown in the top half of Fig. 3. This mode is thus a weak electric linear quadrupole and has a strong magnetic dipole-like resonance, making it particularly suited for applications such as wireless inductive charging^16^. The radiation from these two lowest order modes is almost entirely linearly polarized, in contrast to the elliptical or circular polarization typically associated with conventional single-handed helices.

## Pitch variation of single-handed and Ambihelix structures

**Fig. 4:**
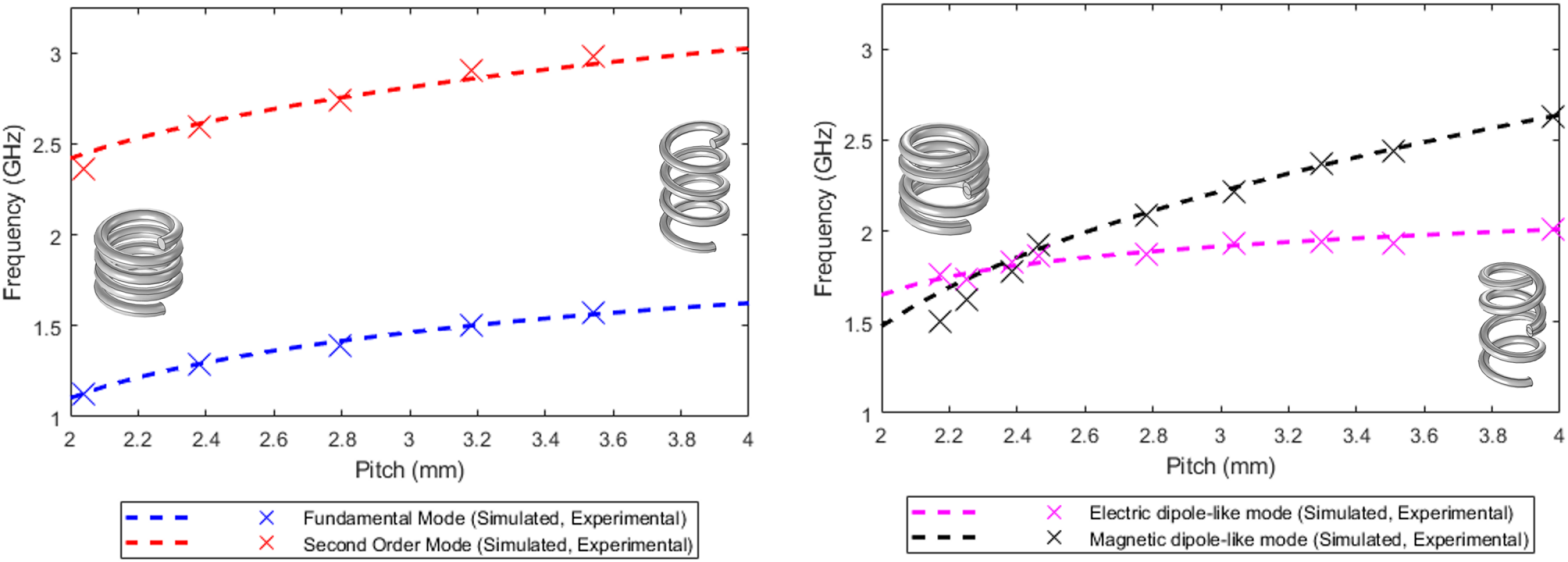
Simulated and experimental pitch dependence of the two lowest order resonant frequencies for $R_\textrm{W}$ = 0.85 mm single handed four turn helix *R* = 4.0 mm (Left) and two left two right turn ambihelix *R* = 4.3 mm , *V* = 0.5 mm (Right).

For comparative analysis, we obtained simulated and experimental results of the impact of pitch variation on the frequencies of the lowest two modes of a four-turn single-handed helix as well as ambihelices, both are shown in Fig. 4.

The two lowest resonant mode frequencies of a single-handed helix have a strong pitch dependence primarily due to the change of inductance with pitch. The naive expression for the inductance of a single-handed N-turn helix is $L = \mu _0 \frac{\pi N R^2}{P}$^18^, which implies a $\sqrt{P}$ dependence of resonant frequency, ignoring capacitance as this only causes a significant shift to the resonant frequency when the wire diameter approaches the pitch. This implies that the resonant frequency of the single-handed helix’s lowest-order mode should change by a factor of ($\sqrt{2}$) when the pitch is doubled, this is close to the factor of $\frac{1.62 }{1.1 }= 1.47$ obtained in the modelling (see Fig. 4). The pitch dependence of the single-handed second-order mode is weaker as the magnetic fields in each half are opposed, which will reduce the inductance, modelling gives a factor of 1.25.

Modelling the electric dipole-like mode of the ambihelix gives a factor of 1.22, when the pitch is doubled. This is close to the 1.25 factor for the single-handed helix second-order mode, as both have opposing magnetic fields in the two halves.

The resonant frequency of the magnetic dipole-like mode of the ambihelix shows a significantly stronger dependence on pitch with a factor of 1.78 for a doubling of the pitch. For this mode there is zero current at the mid-point of the ambihelix so this structure behaves for this mode similarly to two distinct resonators. Not surprisingly this mode has nearly the same frequencies as the magnetically in-phase coupled mode of two vertically aligned, opposite handed helices of two turns, with a small gap between them ($\ll P$). Thus the resonant frequency for this mode is determined by the inductance between each turn as with the other modes and also the coupled inductance between the two halves^19^.

More significantly for the ambihelix the order in frequency of the electric and magnetic dipole-like modes reverses at *P* = 2.3 mm where the modes cross in frequency. This behaviour is not observed in simpler structures, such as the single-handed helix, where the second mode is essentially a harmonic of the fundamental and cannot coincide. This ability to change the geometry and adjust the frequencies of the modes independently gives potential for tailored performance in specific applications such as requiring two specific strong linearly polarised resonances from a single structure.

## Conclusion

The study of ambihelices show that they support distinct electric dipole-like and magnetic dipole-like modes, which can be shifted and even interchanged in frequency by simply changing their pitch. The observed behaviour underscores the interplay between geometry and electromagnetic properties in these structures. By varying the pitch, we can effectively control which resonance mode operates at which frequency, offering a pathway towards tailoring and optimizing the performance of ambihelix structures for specific applications. This versatility holds significant promise for the development of re-configurable and adaptive electromagnetic devices. Also highlighted is the fact that two strong linearly polarised resonances can be obtained from a single ambihelical structure unlike elliptical polarisation from a single handed helix. Moreover, the ability to dynamically switch between electric and magnetic dipole modes through simple geometric adjustments opens avenues for exploring novel functionalities and innovative designs in the realm of metamaterials, antennas, and beyond. In addition the simple four-turn ambihelix exhibited here is only one of the many variants which may be explored.

We also acknowledge financial support from the Defence Science and Technology Laboratory (Dstl) (Contract No: DSTLX1000147175). Alex Powell recognises funding from a Royal Academy of Engineering research fellowship .

## Methods

**Fig. 5:**
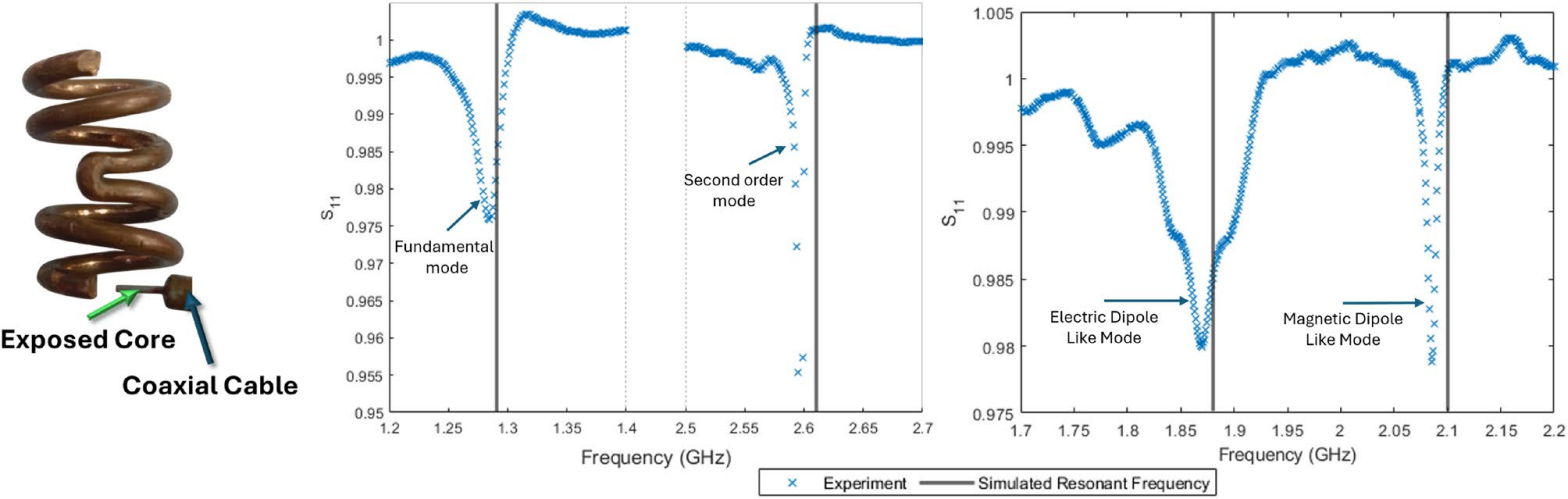
Experimental set up (left) and measured $S_{11}$ response (normalised to the probe in isolation) for $R_\textrm{W}$ = 0.85 mm for a uniform four turn left handed *P* = 2.38 mm, *R* = 4.0 mm (middle) and a two left two right turn ambihelix *P* = 2.78 mm, *R* = 4.3 mm , *V* = 0.5 mm (right) both excited using a short electric dipole. Vertical lines are predicted resonant frequencies from models.

All helical samples were formed by wrapping $R_\textrm{W}$ = 1.7 mm uncoated copper wire around a cylinder of 6.3 mm diameter. For the ambihelices a V-shaped junction (hairpin) was introduced to the winding to allow the creation of two sections of different handedness. The ambihelix has a slightly larger radius than the single-handed helix due to the practical difficulty of wrapping the V-shaped junction, making it less tightly wound. All the above simulated results use *R* = 4.0 mm for comparison between single handed and ambihelical two lowest order modes. Further ambihelix simulated and experimental data use the *R* = 4.3 mm to match the experimental geometry. After removing a helix from the cylinder, the resonances were recorded by monitoring the amplitude of the signal ($S_{11}$) of a coaxial probe connected to port 1 of a vector network analyser when the probe is placed 1 mm from the helical sample wire end, as shown in the top section of Fig. 5, within an anechoic chamber. $S_{11}$ is a scattering parameter that measures how much of an incoming signal is reflected back from a device or structure when a signal is applied at port 1. The plotted data represents the frequency response of the helical sample in proximity to the probe with the response of the probe in isolation subtracted, isolating the influence of the sample. The probe is made of a stripped section of rigid copper coaxial cable with a 2 mm copper exposed core protruding from the cable end. To minimize the effect of the probe on the mode it was designed with a resonant frequency considerably higher (around 20 GHz) than those of the samples, to stop any significant resonant mode overlap between the sample and probe.

To explore the effect of changing pitch on the resonances, helices were initially produced with the lowest feasibly manufacturable pitch, then Mylar$\circledR$ spacers were inserted between the turns in the samples to incrementally increase the pitch and then subsequently removed. The overall wire length and helix radius remains fixed so there is a slight reduction in the number of turns as the pitch is increased. Although this has some effect it is minimal at the pitches we investigate as the change in number of turns is less than $5 \%$.

The left section of Fig. 5 shows the two lowest-order resonances of a four-turn single-handed helix, while the right shows the two lowest-order modes of the ambihelix. The dips in the $S_{11}$ response show that signal is not being returned, corresponding to the structure resonating at that frequency. The single-handed helix results show two modes one at approximately double the frequency of the first as is expected for a harmonic, with the Q-factor of the second mode being much larger, due to the weaker radiation efficiency of the quadrupole compared to the dipole. (At certain points the $S_{11}$ goes above one which is due to the data being normalised to the probe in isolation so at these points more power returns to the VNA than would for an isolated probe.)

The expected lowest-order modes of the ambihelix are a strong electric dipole-like mode and a strong magnetic dipole-like mode. Further, the magnetic dipole-like mode will be much more weakly coupled to free space than the electric dipole-like mode, resulting in a significantly higher Q-factor, as is typical across many structures^20^. This difference in Q-factors allows the resonances to be readily identified from the $S_{11}$ data. It is also notable that they are much closer in frequency than the two lowest order modes of the single handed helix. This is particularly significant because, as we adjust specific parameters, the modes can be made to coincide in frequency or swap order, as shown in the next section and illustrated in Fig. 4.

The difference in Q-factors for both the ambihelix and single handed helices was observed in the simulated results as well highlighting the ability to use them to distinguish modes. Although due to the coupling in method used in the experiential work as opposed to the eigenmodel simulation a direct numerical comparison is impractical.

A slight shift from the FEM prediction of the resonant frequencies is observed. We attribute this to discrepancies in reproducing the exact geometry of each sample. There are also some oscillations observed in the data not associated with the resonances arising from reflections in the cable but these are easily discerned from the resonant modes.

## Data Availability

The data that support the findings of this study are available from the corresponding author upon reasonable request
